# PD-L1 promotes tumor growth and progression by activating WIP and β-catenin signaling pathways and predicts poor prognosis in lung cancer

**DOI:** 10.1038/s41419-020-2701-z

**Published:** 2020-07-06

**Authors:** Wendan Yu, Yijun Hua, Huijuan Qiu, Jiaojiao Hao, Kun Zou, Zongjuan Li, Sheng Hu, Ping Guo, Manyu Chen, Silei Sui, Yuqing Xiong, Fengzhou Li, Jianjun Lu, Wei Guo, Guangyu Luo, Wuguo Deng

**Affiliations:** 1 Institute of Cancer Stem Cells & The First Affiliated Hospital, Dalian Medical University, Dalian, China; 2 Sun Yat-sen University Cancer Center, State Key Laboratory of Oncology in South China, Collaborative Innovation Center of Cancer Medicine, Guangzhou, China; 3 Vagelos College of Physicians and Surgeons, Columbia University, New York, NY USA; 4 The First Affiliated Hospital, Sun Yat-sen University, Guangzhou, China

**Keywords:** Drug development, Lung cancer

## Abstract

PD-L1 is overexpressed in tumor cells and contributes to cancer immunoevasion. However, the role of the tumor cell-intrinsic PD-L1 in cancers remains unknown. Here we show that PD-L1 regulates lung cancer growth and progression by targeting the WIP and β-catenin signaling. Overexpression of PD-L1 promotes tumor cell growth, migration and invasion in lung cancer cells, whereas PD-L1 knockdown has the opposite effects. We have also identified WIP as a new downstream target of PD-L1 in lung cancer. PD-L1 positively modulates the expression of WIP. Knockdown of WIP also inhibits cell viability and colony formation, whereas PD-L1 overexpression can reverse this inhibition effects. In addition, PD-L1 can upregulate β-catenin by inhibiting its degradation through PI3K/Akt signaling pathway. Moreover, we show that in lung cancer cells β-catenin can bind to the WIP promoter and activate its transcription, which can be promoted by PD-L1 overexpression. The in vivo experiments in a human lung cancer mouse model have also confirmed the PD-L1-mediated promotion of tumor growth and progression through activating the WIP and β-catenin pathways. Furthermore, we demonstrate that PD-L1 expression is positively correlated with WIP in tumor tissues of human adenocarcinoma patients and the high expression of PD-L1 and WIP predicts poor prognosis. Collectively, our results provide new insights into understanding the pro-tumorigenic role of PD-L1 and its regulatory mechanism on WIP in lung cancer, and suggest that the PD-L1/Akt/β-catenin/WIP signaling axis may be a potential therapeutic target for lung cancers.

## Introduction

Lung cancer is one of the most common and aggressive cancers with the highest incidence and lethality^1^. At present, the treatment strategies for lung cancer include chemotherapy, radiotherapy, molecular targeting therapy, and immunotherapy. However, chemotherapy and radiotherapy have limited efficacy^2^, and molecular targeting therapy and immunotherapy are in progress^3,4^.

More and more studies have shown that tumor-induced immune suppression is responsible not only for tumor progression^5–7^, but also for inhibition of anti-tumor treatment^8^. One of the major immune evasion molecules is programmed death-ligand 1 (PD-L1, CD274), which can inhibit the activation of T cells^9^. Previous studies have shown that PD-L1 plays an important role in pregnancy maintenance. PD-L1 blockage can increase fetal resorption and Tfr cells but does not affect Tfh/Tfr ratio and B-cell maturation during allogeneic pregnancy^10,11^. PD-L1 is also associated with tumor growth and progression. It is highly expressed in various cancers, such as melanoma, non-small cell lung cancer (NSCLC), renal cell carcinoma, hand and neck cancer, breast cancer, pancreatic cancer, and ovarian cancer^12–17^. More studies about tumor-intrinsic role of PD-L1 in promoting cancer initiation, metastasis, development, and chemo, or radiotherapy resistance are now emerging^18–21^. However, the role of PD-L1 in regulating lung cancer growth and progression and its underlying mechanism remains unclear.

WIP is known as WIPF1 (WASP-interacting protein family member 1), which has been reported to form WIP/WASp complex for immune response and take part in cancer invasion and metastasis^22,23^. WIP controls tumor growth through stabilization of the YAP/TAZ complex via forming the endocytic/endosomal system in glioma^24^. WIP also promotes PDAC cell invasion, metastasis and predicts poor prognosis in PDAC patients^25^. In addition, it has shown that WIP induces EMT in lung cancer A549 cells by regulating RhoA^26^. However, the function of WIP and its mechanism of action in lung cancer have not been elucidated.

In this study, we investigated the role of PD-L1 in the regulation of cell proliferation, migration, invasion, and tumor growth in lung cancer cells and mouse model. We also discovered and identified the downstream targets of PD-L1, and showed that PD-L1 functioned as a tumor-promoting factor mainly through regulating the PI3K/Akt, Erk, EMT, β-catenin, and targeting the WIP signaling pathways in lung cancer. We also analyzed the transcriptional regulation of WIP and identified its role in the PD-L1-mediated lung cancer growth. Moreover, we assessed the correlation of PD-L1 and WIP expression and their clinical significance in lung cancer patients. Taken together, our findings have demonstrated that PD-L1 regulates tumor growth and progression by targeting the WIP and β-catenin signaling pathways.

## Materials and methods

### Cell lines and cell culture

Human NSCLC cell lines H1299, A549, H460, H358, HCC827, H322, and HLF cell were purchased from American Type Culture Collection (ATCC). HLF was cultured in Dulbecco’s modified Eagle’s medium (Gibco) supplemented with 10% fetal bovine serum. H1299, A549, H460, H358, and H322 were cultured in RPMI-1640 medium supplemented with 10% fetal bovine serum. All referred cell lines were cultured in a humidified incubator containing 5% CO_2_ at 37 °C.

### Plasmid construction and stable PD-L1 overexpression

The pmCherry C1 human WIP plasmid was purchased from Addgene (#29573). To generate the pcDNA3.1-β-catenin plasmid, we amplified the full-length β-catenin gene by RT-PCR from H1299 cell cDNA and cloned it into pcDNA3.1 vector (V790, invitrogen, USA). To generate the WIP gene promoter-driven luciferase reporter plasmid, the WIP gene upstream sequences (−1691 to +28, −266 to +28) were cloned into PGL3-basic plasmid (Promega, Madison, WI, USA) respectively. The pLX304-Blast V5-PD-L1 plasmid and Empty Vector (#OHS6895, Thermo, USA) were transfected into HEK-293T cells with lenti-HIV expression packaging kit (LT001, GeneCopoeia, Guangzhou, China) to produce lentivirus. At 48 h after transfection, lentivirus was used to transduct H1299 and A549 cells. After 72 h, 1 µg/ml or 8 µg/ml Blastcidin S was used for selecting drug-resistant cells.

### siRNAs and shRNAs

The siRNA (shRNA) sequences used in this study are as follows:

PD-L1 siRNA-1, 5′-GGCACAUCCUCCAAAUGAATT-3′ and PD-L1 siRNA-2, 5′-GAAGCAAAGUGAUACACAUTT-3′; WIP siRNA-1, 5′-CCAAGACCCAUUCAAUCAATT-3′ and WIP siRNA-2, 5′-GGAUCCAACCGAAGAGAAATT-3′; β-catenin siRNA, 5′-GGACACAGCAGCAAUUUGUTT-3′; All siRNAs were synthesized by GenePharma (GenePharma, Suzhou, China). PD-L1 shRNA #4, 5′-TGAAGAAAGATGGAGTCAA-3′ and PD-L1 shRNA #5, 5′-GTAGCAATATGACAATTGA-3′; WIP shRNA #3, 5′-CCAAAAGTTATCCCAGCAA-3. The pGIPZ lentiviral shRNA vector was used to express all shRNAs in H460 cells.

### RT-PCR

Total RNA was extracted using the Trizol reagent (15596-026; Invitrogen, USA). The cDNAs were synthesized using TransScript One-Step gDNA Removal and cDNA synthesis SuperMix (Transgene, Beijing, China). The primer sequences were as follows: PD-L1 forward, 5′-GCTGTTGAAGGACCAGCTCT-3′ and PD-L1 reverse, 5′-TGGAGGATGTGCCAGAGGTA-3′; CTNNB1 forward, 5′-ATGACTCGAGCTCAGAGGGT-3′ and CTNNB1 reverse, 5′-ATTGCACGTGTGGCAAGTTC-3′; WIP forward, 5′-CCCTCCTCCTCCTCAGAACA-3′ and WIP reverse, 5′-ATTCCGCTGTGGGAGTCTTG-3′; GAPDH forward, 5′-AATCCCATCACCATCTTCC-3′ and GAPDH reverse, 5′-CATCACGCCACAGTTTCC-3′.

### Antibodies and western blot

The anti-PD-L1 (ab121545) was purchased from Abcam (Cambridge, MA, USA). The anti-p-S6 (S_235/236_)(4858T), anti-β-catenin (8480S), anti-Akt (4691S), anti-p-Akt (T_308_)(13038T), anti-p-PDK1 (Ser_241_)(3438T), anti-p-GSK3β (9323S), anti-p-P70S6K (T_389_) (9234T), anti-OCT4 (2750S), anti-pErk (4370S), anti-Erk (4695S), anti-CyclinD1 (2978S), anti-p-YAP (13008T), anti-Claudin-1 (13255T), and anti-Histone H3 (4499S) were purchased from Cell Signaling Technology (Danvers, MA, USA). The anti-WIP (sc-271113), anti-β-catenin (sc-7963), anti-MMP-9 (sc-21733), and anti-PCNA (sc-25280) were purchased from Santa Cruz (Santa Cruz, CA, USA). The anti-actin (20536-1-AP), anti-GAPDH (10494-1-AP), anti-ki67 (19972-1-AP), anti-E-cadherin (20874-1-AP), anti-N-cadherin (22018-1-AP), anti-CD44 (15675-1-AP), anti-Bax (50599-2-lg), anti-Bcl-2 (12789-1-AP), and anti-YAP (13584-1-AP) were purchased from Proteintech (Wuhan, China). The protein bands were detected by ECL according to the manufacturer’s instructions.

### Dual-luciferase assay

The WIP promoters were amplified using H460 gDNA as template and cloned into the pGL3-basic vector. H1299 cells were seeded in six-well plates. One the next day, the cell were transfected with Empty Vector or pcDNA3.1-β-catenin plasmid. After 24 h, WIP promoter-driven luciferase plasmids and pRL-TK were also co-transfected into the above cells. Afterb 24 h, cells were harvested. Luciferase activity was measured using the dual-luciferase reporter assay system (E1910, Promega).

### MTT assay

Cells plated in 96 well plates were transfected with plasmid or siRNAs. After 48 h, 10% MTT (5 mg/ml) were added to the cells with continuous culture for 4 h.The OD value was measured at 492 nm.

### Colony formation assay

Cells infected by lentivirus contain Empty/PLX304-Blast-V5-PD-L1 or scramble/specific shRNAs were trypsinized into single cell and seeded in 6-well plates (1000 cells/well) with continuous culture for 10–14 days. Then the cells were washed with PBS and fixed with the buffer (Methanol: Glacial Acetic Acid: Water = 1:1:8) for 10 min, and stained with 0.1% crystal violet for 15 min. After wash with PBS, cell colonies that contained more than 50 cells were counted and photographed.

### Wound-healing assay

Cells were seeded in six-well plate and grown to 70–80% confluence. Then the cells were transfected with PD-L1 or WIP siRNAs for 24 h and scraped in a straight line to create a “scratch”. The wound gaps were photpgraphed at different intervals and quantified the migration rate of the cells.

### Transwell invasion assay

The transwell invasion assay was performed using 24-well chemotaxis chambers (Corning, CA, USA, Cat#:3422). The upper chambers were coated with a mixture of serum-free medium and Matrigel (6:1; BD Biosciences, cat#:356234). The cells were washed twice with PBS and resuspended in 100 µl serum-free medium then added into upper chamber. The lower chambers were filled with 500 µl medium containing 20% FBS. After incubated at 37 °C for 48 h or 72 h, the cells located at the underside of the chamber were washed twice with PBS, fixed with 4% paraformaldehyde for 10 min and stained with 0.1% crystal violet for 15 min, then washed three times with PBS and dried off. Images of the cells were recorded by invert microscope and the invasiveness of cells was calculated by total number of cells in three randomly selected fields.

### ChIP assay

PD-L1 stable overexpression H1299 cells were transfected with β-catenin siRNA. After 48 h transfecteion, cells in 10-cm dishes were fixed with 1% formaldehyde for 10 min at RT, then 10% 1.25 M glycine was added into the medium to end the crosslink. The cells were washed with cold PBS for three times and then scraped and harvested in PBS containing 0.5 mM PMSF, 1 mM Na_3_VO_4_, 0.1 mM DTT,1 mM Leuptin, 2.5 mM β-glycerophosphate, 0.5 M NaF. Wash the cells with PBSI for three times. The cell pellets were resuspended with 500 µl IP buffer (SDS buffer: Triton Dilution buffer = 2:1) and sonicated five times for 5 s with 7 s pause each time, then centrifuged at 14,000 × *g* for 20 min at 4 °C and transferred the supernatants into a new tube. Twenty-five microliters protein A/G agarose beads (Santa Cruz Biotechnology) were mixed with 1 mg total proteins and rotated for 30 min at 4 °C. After centrifugation for 15 min at full speed, the chromatin supernatant was immunoprecipitated overnight with 2 µg antibodies against β-catenin(Santa Cruz Biotechnology) or anti-mouse IgG. Then 45 µl protein A/G agarose beads were added into the mixture and rotated for 8 h at 4 °C. The pellets were washed for 5 min with the following buffers: Mixed wash buffer twice, Buffer 500 twice, Licl/detergent wash buffer twice, and TE buffer twice. The beads were reversely cross-linked by heating at 65 °C overnight in 1% SDS, 0.1 M NaHCO_3_ buffer. After brief centrifuge, the supernatant was digested with 250 µl proteinase K solution at 37 °C for 2 h. DNA was finally extracted by phenol/chloroform/Isoamyl alcohol extractions and used as DNA templates to amplify the specific WIP promoter region. The primers used for PCR was as follow: Forward primer, 5′-TCTCCCTTCCCCCTTCAG-3′; Reverse primer, 5′-TCTCGAGTTCCCCTGCTGTC-3′.

### DNA pulldown assay

Four hundred micrograms nuclear proteins were mixed with 0.8 µg double-strand biotinylated WIP promoter probe and 50 µl streptavidin agarose beads in 400 µl prepared PBSI buffer containing 0.5 mM PMSF, 1 mM Na_3_VO_4_, 0.1 mM DTT, 1 mM Leuptin, 2.5 mM β-glycerophosphate, 0.5 M NaF, then gently rotated at RT overnight. The supernatant was discarded and the beads were washed with 300 µl PBSI five times. The pellet was resuspended with 40 µl 1× loading buffer and boiled at 100 °C for 10 min. The supernatant was analyzed by western blot.

### Patient tissue preparation and tissue microarray assay

Hmuan lung adenocarcinoma tissues from six patients were obtained at the First Affiliated Hospital of Dalian Medical University from January to December 2015 according to the 8th Edition International Union Against Cancer/American Joint Committee on Cancer TNM classification. Patients who received chemotherapy or radiotherapy prior to the operation were excluded. The study was approved by the Medical Ethical Committees of the First Affiliated Hospital of Dalian Medical University. All patients were informed of the study. The human lung adenocarcinoma tissue microarrays were purchased from Outdu Biotech Company (Shanghai, China) containing 92 lung adenocarcinoma tissues and paired normal lung tissues (cat# HLugA180Su03), all the clinicopathological information can be downloaded from website (Http://www.superchip.com.cn). The protein expression levels of PD-L1 and WIP were detected by IHC assay and analyzed according to the staining level of tissue microarrays.

### Immunohistochemistry staining

The tissue were fixed by 4% paraformaldehyde, washed with PBS three times, transferred to 70% ethanol and then embedded in paraffin according to standard procedures. After dewaxed with graded ethanol solution and antigen retrieval, the tissue was stained using Streptavidin Peroxidase IHC assay kit (SP-9000, ZSGB-Bio, China). The antibodies against PD-L1 (Abcam, dilution 1:200), β-catenin (Santa Cruze, dilution 1:50), WIP (Santa Cruze, dilution 1:50), p-S6 (CST, 1:200), PCNA (Proteintech dilution 1:50), and Ki67 (Proteintech, dilution 1:50) were used. Immunostaining was evaluated by two pulmonary pathologists using a blind protocol design. For each specimen, the total score of PD-L1 and WIP expression was calculated as staining intensity (negative staining: 0 point; weak staining: 1 point; moderate staining: 2 point; and strong staining: 3 point) multiplied by the point of the percentage of stained cells (positive cells ≤ 25% of the cells: 1 point; 26–50% of the cells: 2 point; 51–75% of the cells: three point; ≥75% of the cells: 4 point). The positive controls of PD-L1 and WIP were set up according to lung cancer from the protein-atlas website (Http://www.proteinatlas.org).

### In vivo xenograft mouse model

All animal experiments were performed according to the animal licence protocol approval by Animal Care and Ethics Committee of Dalian Medical University. The 5 week-old male BALB/c-nude mice were purchased from Beijing Vital River Laboratory Animal Technology. For xenograft tumor formation, the mice were randomly divided into four or three groups (*n* = 6), EV + NC, EV + shWIP, PD-L1 + NC, PD-L1 + shWIP or shCtrl, PD-L1 sh4, PD-L1 sh5. Each mouse was subcutaneously injected with 1 × 10^7^ cells in 200 µl PBS. Tumor volume was measured after 10 days after injection. The tumor volume was calculated as *V* = (width^2^ × length)/2 and the data was recorded every 2 days within 10 days. Mice were sacrificed and tumors were taken from mice for weighting and photographing, partial tissues were transferred to liquid nitrogen immediately and lysed for western blot, other tissues were fixed in 10% formalin for IHC assay.

### Statistical analysis

Each experiment was done three times and the results were presented as the mean ± SE. Student’s *t* test was used to compare two independent groups of data. The correlation between PD-L1 and WIP expression was evaluated by Pearson Chi-squared test. *P* < 0.05 was considerate to be significant.

## Results

### PD-L1 regulates lung cancer cell growth in vitro and in vivo

**Fig. 1:**
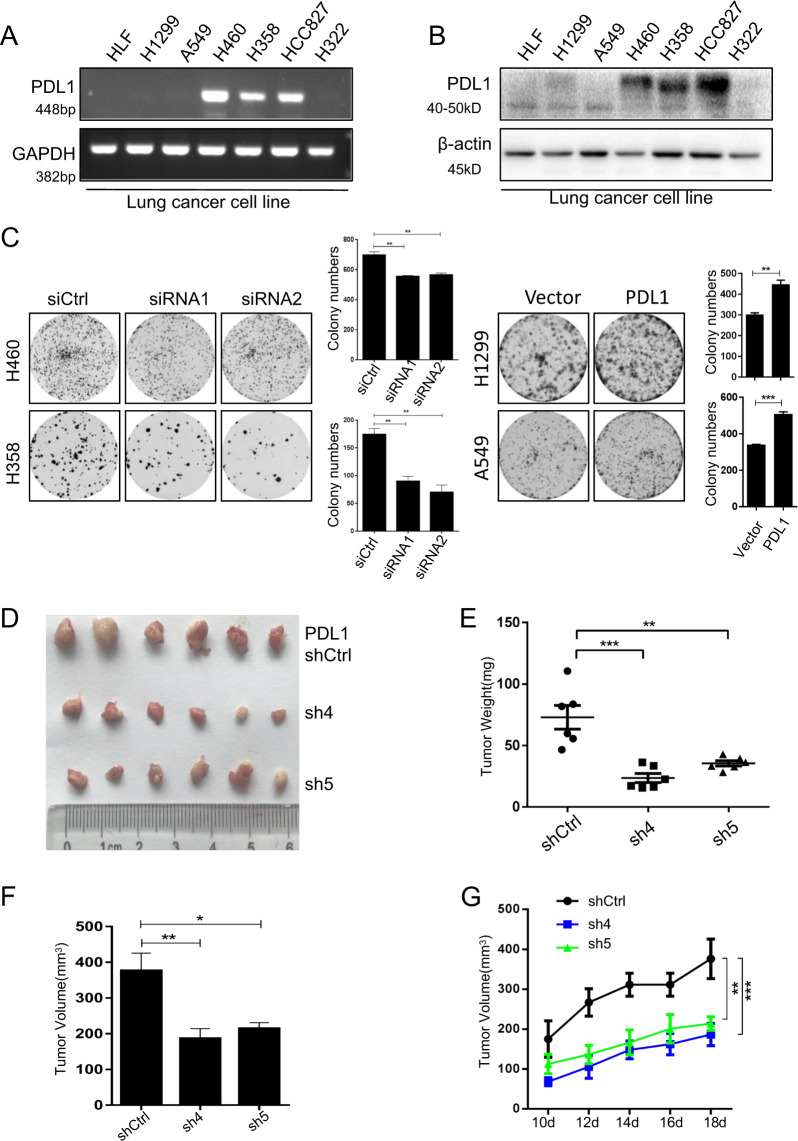
PD-L1 promotes lung cancer cell growth in vitro and in vivo. **a** PD-L1 mRNA expression in different human lung cancer cell lines was analyzed by RT-PCR. **b** PD-L1 expression in human lung cancer cells was detected by western blot. **c** Colony formation assay in human lung cancer cells with PD-L1 knockdown or overexpression. **d** The xenografts were harvested at 18 days after injection and the morphology of tumor xenografts from each nude mouse was photographed. **e** The tumor weight of each nude mouse was measured. **f** The tumor volume of each mouse from different groups was measured and calculated as Volume = (Width^2^ × length)/2. **g** Growth kinetics (mean ± SD) of PD-L1-sh4, PD-L1-sh5 versus the negative control in BALB/c-nude mice (*n* = 6). The data are presented as mean ± SD of three independent tests. *P < 0.05, **P < 0.01, ***P < 0.001.

PD-L1 plays an important role in tumor immune evasion. To investigate the tumor-intrinsic role of PD-L1 and its underlying mechanisms, we examined the effect of PD-L1 on lung cancer cell growth in vitro and in vivo. We first detected PD-L1 mRNA and protein levels in human lung fibroblast (HLF) cell lines and six NSCLC cell lines (H1299, A549, H460, H358, HCC827, and H322) by RT-PCR (Fig. 1a) and western blot (Fig. 1b). PD-L1 was lowly expressed in fibroblasts, H1299, A549, and H322 cell lines, but highly expressed in H460, H358, and HCC827 cell lines. We then knocked down PD-L1 expression in H460 and H358 cells by PD-L1 specific siRNAs and overexpressed PD-L1 in H1299 and A549 cells (Supplementary Fig. S1A). The results showed that PD-L1 knockdown significantly inhibited colony formation and cell viability in H460 and H358 cells, while PD-L1 overexpression increased colony formation and cell viability in H1299 and A549 cells (Fig. 1c, Supplementary Fig. S1B).

Next, we confirmed the pro-tumorigenic role of PD-L1 in human NSCLC in vivo. We generated the stable PD-L1 shRNA knockdown H460 cells and tested the effect of PD-L1 knockdown on tumor growth in immunocopromised BALB/c-nude mice. Compared with the shRNA control group, knockdown of PD-L1 by shRNA (sh4 or sh5) markedly suppressed tumor growth in mice administrated with stable PD-L1 knockdown H460 cells, resulting in significant reductions in tumor size, weight and volume (Fig. 1d–g). Western blot and immunohistochemical assay showed that PD-L1 was knocked down by specific shRNA (Supplementary Fig. S1C, D). These results demonstrate that PD-L1 played an intrinsic role in regulating tumor formation and progression.

It has been reported that PD-L1 expression was response to EMT/CSC-like phenotypes in bladder cancer cells and breast cancer stem cells (CSCs)^27,28^. To investigate if PD-L1 was involved in tumor stemness character and cell apoptosis, we overexpressed and knocked down PD-L1 in lung cancer cells, and found that PD-L1 overexpression or knockdown did not affect the expression of the CSC-related markers (CD44, OCT4) or the apoptosis-related molecules (Bax and Bcl-2) (Supplementary Fig. S2A).

### PD-L1 promotes lung cancer cell migration and invasion via EMT signaling

**Fig. 2:**
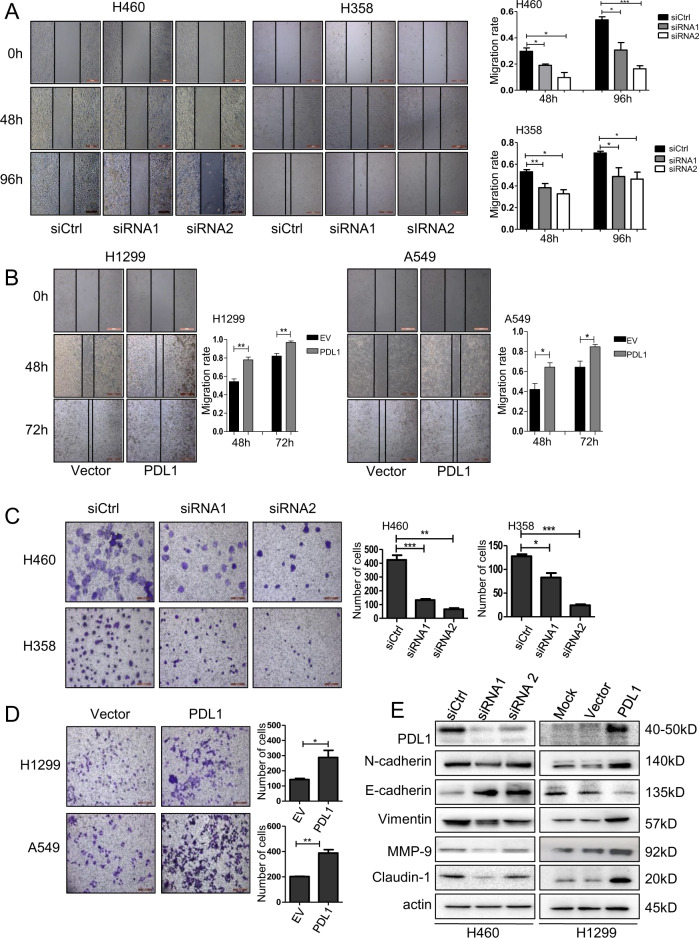
PD-L1 promotes lung cancer cell migration and invasion via EMT signaling. H460 and H358 cells were transfected with PD-L1 siRNAs, and H1299 and A549 cells were transfected with PD-L1-OE plasmid. After 48 h, **a**, **b** Cell migration was analyzed by cell wound-healing assay and the migration rate was calculated; **c**, **d** Cell invasion assay was performed, and the number of invasion cells was counted. The data are presented as mean ± SD of three independent tests. *P < 0.05, **P < 0.01, ***P < 0.001; **e** The expression levels of PD-L1, N-cadherin, E-cadherin, Vimentin, MMP-9, Caludin-1 and β-actin were analyzed by western blot.

PD-L1 promotes EMT in esophageal cancer^13^. We next analyzed the effect of PD-L1 on NSCLC cell migration and invasion, and showed that knockdown of PD-L1 by siRNAs significantly inhibited cell migration and invasion compared with the siRNA control in H460 and H358 cells (Fig. 2a, c), whereas overexpression of PD-L1 promoted cell migration and invasion (Fig. 2b, d). To further verify the role of PD-L1 in regulating cell migration and invasion, we evaluated the effect of PD-L1 on the EMT signaling, and found that PD-L1 effectively modulated the expression of the EMT-related molecules. Knockdown of PD-L1 suppressed the expression of N-cadherin, Vimentin, MMP-9 and Claudin-1 but upregulated E-cadherin expression in lung cancer cells (Fig. 2e). In contrast, PD-L1 overexpression upregulated the expression of N-cadherin, Vimentin, MMP-9, Claudin-1 but inhibited E-cadherin expression (Fig. 2e). These results indicated that PD-L1 promoted lung cancer cell migration and invasion by targeting EMT signaling.

### WIP is a downstream target of PD-L1 in lung cancer cells

**Fig. 3:**
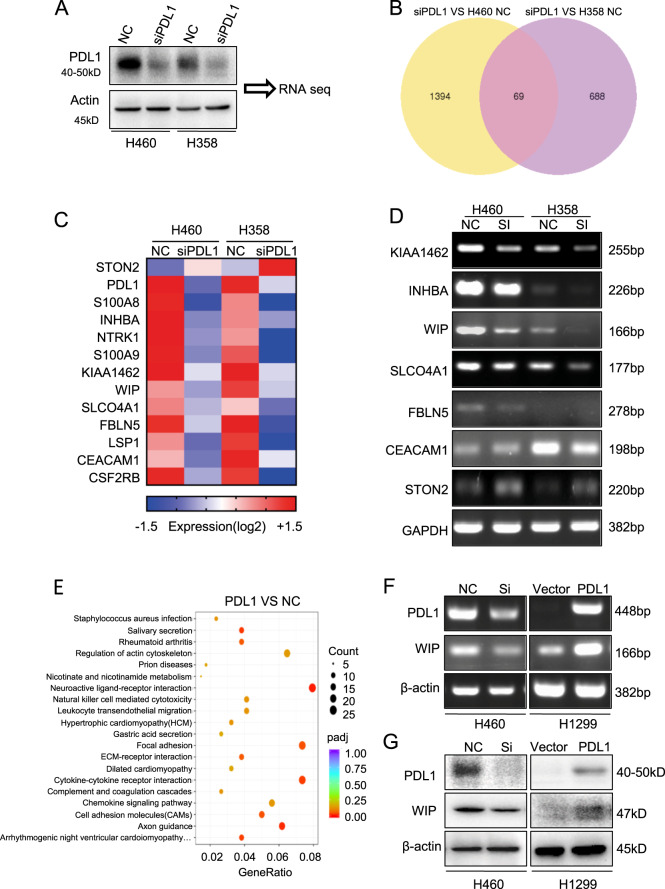
WIP is a downstream molecule of PD-L1. **a** PD-L1 was knocked down by siRNA in H460 and H358 cells. (**b**) Overlap of changed genes affected by PD-L1 siRNA silencing in H460 and H358 lung cancer cells. **c** Heatmap showing the subset of 12 genes identified in H460 and H358 NSCLC cells followed by PD-L1 silencing. **d** The regulation of gene expression by PD-L1 was analyzed by RT-PCR assay in H460 and H358 cells. **e** KEGG analysis showed that genes were enriched in the actin cytoskeleton pathway. **f**, **g** The expression of WIP was detected by RT-PCR and western blot.

To further elucidate the underlying molecular mechanism of PD-L1 in regulating lung cancer tumor growth and progression, we performed RNA-sequencing assay in PD-L1-knocked down cells (Fig. 3a). PD-L1 knockdown significantly regulated the expression of 69 genes in both H460 and H358 cells (Fig. 3b). Furthermore, heat-map analysis demonstrated that PD-L1 knockdown inhibited 11 genes and upregulated 1 gene (Fig. 3c). We then confirmed the effect of PD-L1 on the expression of these genes in H460 and H358 cells by RT-PCR, and found that WIP was regulated most significantly by PD-L1 knockdown (Fig. 3d). KEGG analysis also showed that these genes were enriched in actin cytoskeleton pathway, which included WIP (Fig. 3e). Moreover, we further verified the regulation of PD-L1 on WIP expression in H460 and H1299 cells. The results showed that PD-L1 knockdown inhibited WIP expression at both mRNA and protein levels, whereas PD-L1 overexpression promoted WIP expression (Fig. 3f, g). These results indicate that WIP is a downstream target of PD-L1 in lung cancer.

### PD-L1 activates the PI3K/Akt signaling pathway by stabilizing β-catenin

**Fig. 4:**
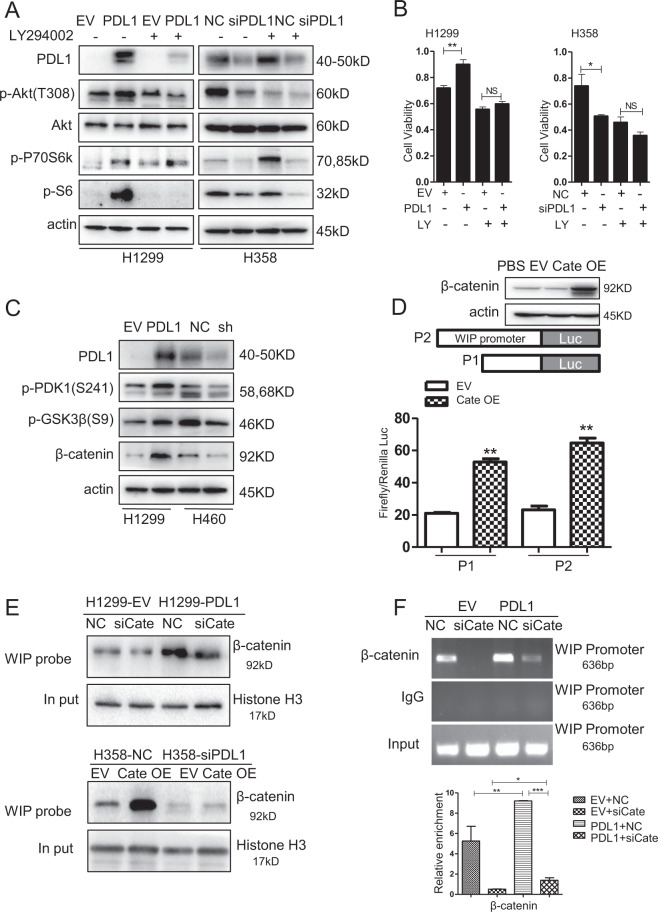
PD-L1 activates PI3K/Akt pathway to promote the β-catenin mediated transcription of WIP. **a** H1299 and H358 cells were pretreated with 10 µM LY294002 for 4 h, and then cells were transfected with PD-L1-OE plasmid or siRNA for 48 h. The expression of p-Akt, Akt, p-P70S6K, p-S6, and β-actin were detected by western blot. **b** H1299 and H358 cells were pretreated with 10 µM LY294002 for 4 h and then transfected with PD-L1-OE plasmid or siRNA for 48 h, and cell viability was measured by MTT assay. **c** Expression of PD-L1, p-PDK1, p-GSK3β, β-catenin were detected by western blotting after 48 h transfection of PD-L1-OE plasmid or siRNA in H1299 and H460 cells. **d** H1299 cells were co-transfected with β-catenin-OE plasmid or empty vector and WIP promoter reporter plasmid. At 48 h after transfection, promoter activity was analyzed using dual-luciferase assay. **e** Binding of β-catenin to the WIP promoter was detected by DNA pulldown assay after PD-L1 overexpressed alone or PD-L1 overexpressed and β-catenin silenced. **f** Binding of β-catenin to the WIP promoter was analyzed by ChIP analysis. For **b**, **d**, **f**, the experiments were repeated three times, and the results were shown as mean ± SD. *P < 0.05, **P < 0.01, ***P < 0.001. NS means no significant differences between two groups. LY means LY294002.

PD-L1 has been shown to augment phosphorylation of S6 ribosomal protein by activating mTOR signaling in melanoma^29^. To determine whether PD-L1 in lung cancer had a similar function, we analyzed the expression of p-S6 and p-P70S6K. We found that treatment of the PI3K inhibitor LY294002 abrogated the PD-L1-mediated upregulation of S6 phosphorylation (p-S6). PD-L1 overexpression also increased the level of p-Akt (T308) (Fig. 4a). In addition, we showed that mTOR inhibitor rapamycin and MEK1/2 inhibitor U0126 could reverse the PD-L1-mediated upregulation in p-S6 expression (Supplementary Fig. S3A, B). To further conform the role of PI3K/AKT and Erk-signaling pathway in PD-L1 mediated proliferation in lung cancer cells, LY294002 and U0126 were used to pretreated H1299 and H358 cells, and followed by transfection with PD-L1 overexpression plasmid or PD-L1 specific siRNA.LY294002, significantly inhibited lung cancer cell proliferation, whereas inhibitor pretreatment followed by PD-L1 overexpression or knockdown did not obviously alter the proliferative promotion or inhibition in lung cancer cells, indicating the essential role of PI3K/AKT in lung cancer proliferation (Fig. 4b). The same studies were used in MEK1/2 inhibitor, U0126 and PD-L1 overexpression or siRNA combinational treatment did not change the cell growth and invasion inhibition, compared with U0126 treatment alone (Supplementary Fig. S3C, D). These results showed that the regulation of lung cancer cells growth mediated by PD-L1 was partially activated via PI3K/AKT/mTOR and ERK pathway.

Considering the crosstalk between PI3K/Akt and GSK3β, we next investigated the effect of PD-L1 on p-GSK3β (Ser9) and β-catenin. We found that PD-L1 overexpression increased β-catenin protein levels by promoting phosphorylation of GSK3β at the Serine 9 (inactive form of GSK3β), which could increase protein stability (Fig. 4c), while the level of β-catenin mRNA was not changed (Supplementary Fig. S4A). These results indicated a post-transcription regulation of PD-L1 on β-catenin.

### PD-L1 promotes the binding of β-catenin on the WIP promoter

We next determined whether β-catenin bound to the WIP gene promoter and regulated WIP transcription in lung cancer cells. The sequence upstream of the WIP gene was analyzed using ALGGEN-PROMO database (Http://alggen.lsi.upc.edu). We found that the WIP gene promoter had potential LEF/TCF-binding sites (Supplementary Fig. S4B). Moreover, luciferase reporter gene assay showed that overexpression of β-catenin activated the WIP promoter activity in H1299 cells (Fig. 4d).

To further test whether β-catenin bound to the WIP promoter, a biotinylated WIP promoter probe was synthesized and pulldown assay was performed. As shown in Fig. 4e, the binding of β-catenin on the WIP promoter was observed. Overexpression of PD-L1 increased the binding activity of β-catenin on the WIP promoter, while PD-L1 knockdown led to remarkable decrease of this binding. Moreover, chromatin immunoprecipitation (ChIP) assay also validated the binding of β-catenin on the WIP gene promoter and the PD-L1-induced promotion of the binding in H1299 cells (Fig. 4f).

### The PD-L1-mediated promotion of cell growth is partially dependent on the WIP signaling

**Fig. 5:**
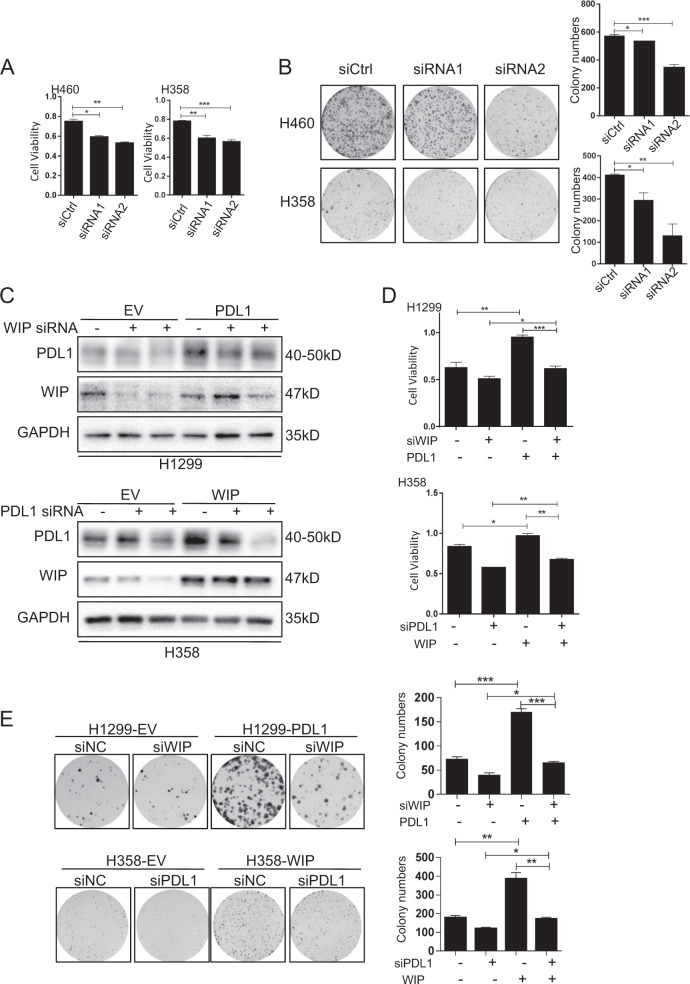
The PD-L1 mediated cell growth promotion is partially dependent on WIP. **a**, **b** H460 and H358 cells were transfected with WIP siRNAs. After 48 h, cell viability and colony numbers were analyzed by MTT and colony formation assays. **c** H1299 cells were transfected with PD-L1-OE and WIP siRNAs, and H358 cells were transfected with PD-L1 siRNAs and WIP-OE plasmid. After 48 h, the expression of PD-L1, WIP, and GAPDH were detected by western blot. **d**, **e** H1299 cells were treated with PD-L1-OE and WIP siRNAs, and H358 cells were treated with PD-L1 siRNA and WIP-OE plasmid. After 48 h, cell viability and colony numbers were analyzed by MTT and colony formation assays. For **a**, **b**, **d**, **e**, the data represent the mean ± SD of three independent experiments, *P < 0.05, **P < 0.01, ***P < 0.001.

We have showed that PD-L1 could transactiviate WIP. We then evaluated the function of WIP in regulating cell growth. As shown in Fig. 5a, b, WIP knockdown by its specific siRNAs significantly suppressed cell viability and colony formation. To investigate the relationship between PD-L1 and WIP, we performed the rescue experiments by overexpressing PD-L1 but knocking down WIP in H1299 cells, and knocking down PD-L1 but overexpressing WIP in H358 cells. As shown in Fig. 5c and Supplementary Fig. S5, WIP knockdown did not affect PD-L1 expression in H1299 cells, but PD-L1 overexpression rescued the WIP siRNA-mediated inhibition of WIP in H1299 cells. Conversely, PD-L1 knockdown suppressed WIP expression, but WIP overexpression did not changed PD-L1 expression level in H358 cells. These results indicated that WIP is a downstream target of PD-L1.To further determine whether PD-L1 promoted cell proliferation through WIP in lung cancer cells, As expected, PD-L1 overexpression significantly reversed the WIP knockdown-mediated inhibitions of cell proliferation and colony formation (Fig. 5d, e), indicating that PD-L1 promoted lung cell growth partially via WIP signaling.

It has been well established that WIP drives tumor progression by stabilizing the YAP/TAZ complex, we speculated that PD-L1 may have effects on YAP in NSCLC cells. As expected, PD-L1 overexpression inhibited the expression of p-YAP but not total YAP. Moreover, PD-L1 overexpression or knockdown also increased or decreased the expression of the β-catenin targeting gene CyclinD1 (Supplementary Fig. S4C). To further validate the effect of PD-L1 on p-YAP, we performed IF assay. The result showed that PD-L1 promoted the translocation of YAP into nucleus (Supplementary Fig. S4D).

### PD-L1 promotes tumor growth via upregulating WIP in human lung cancer mouse model

**Fig. 6:**
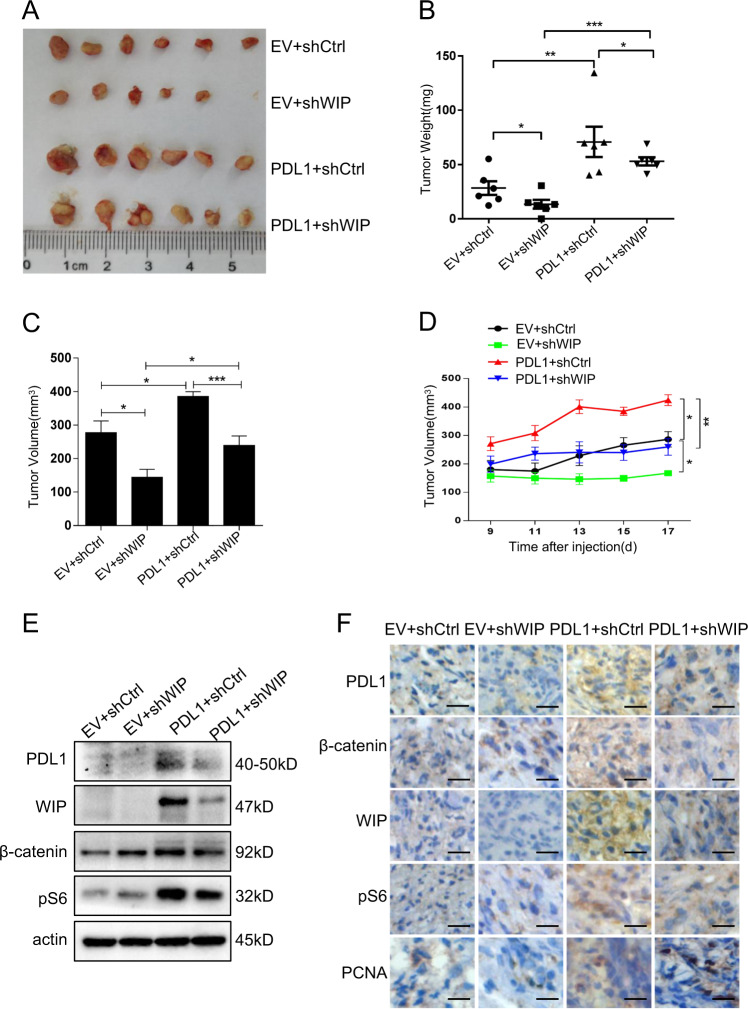
PD-L1 promotes tumor growth by upregulating WIP in human lung cancer mouse model. **a** The morphology of tumor xenografts from each mouse was photographed. **b**, **c** The mouse of each group was sacrificed, the tumor weight and tumor volume were measured. **d** Tumor diameters of each nude mouse from different group were measured at a regular interval of 2 days after 9 days of injection. Tumor volume = (Width^2^ × length)/2. **e** The expression level of PD-L1, β-catenin, WIP, and p-S6 within tumor xenografts in each group of nude mice were detected. **f** The expression of PD-L1, β-catenin, WIP, p-S6, and Ki67 in tumor tissues was detected by immunohistochemistry staining. *N* = 6 mice/group. Scale bars = 50 μm. Original magnification: ×40. The level of significance was indicated by *P < 0.05, **P < 0.01, ***P < 0.001.

To verify the regulation of PD-L1/WIP signaling on lung cancer growth, we also investigated the effect of PD-L1 on tumor growth in a human lung cancer mouse model. We implanted the human A549 cell lines into BALB/c-nude mice to generate human lung cancer xenografts. We first established the A549 cell lines with stable PD-L1 overexpression, or the cells with stable PD-L1 overexpression and WIP shRNA knockdown. After 2 weeks injection of the A549 cells with different expression of PD-L1 and WIP, mice were sacrificed and tumor weight and volume were evaluated. The results showed that overexpression of PD-L1 significantly promoted tumor growth, whereas knockdown of WIP reduced tumor growth (Fig. 6a–d). Western blot analysis for tumor tissues showed that PD-L1 overexpression increased expression of β-catenin, WIP and p-S6 (Fig. 6e). Moreover, the immunohistochemical analysis of tumor tissues similarly revealed that PD-L1 overexpression increased the expression of β-catenin, WIP and p-S6 (Fig. 6f). These in vivo data confirmed that PD-L1-mediated tumor growth was realized through the WIP signaling in lung cancer.

### PD-L1 is positively correlated with WIP expression in human lung adenocarcinoma tissues and predicts poor prognosis

**Fig. 7:**
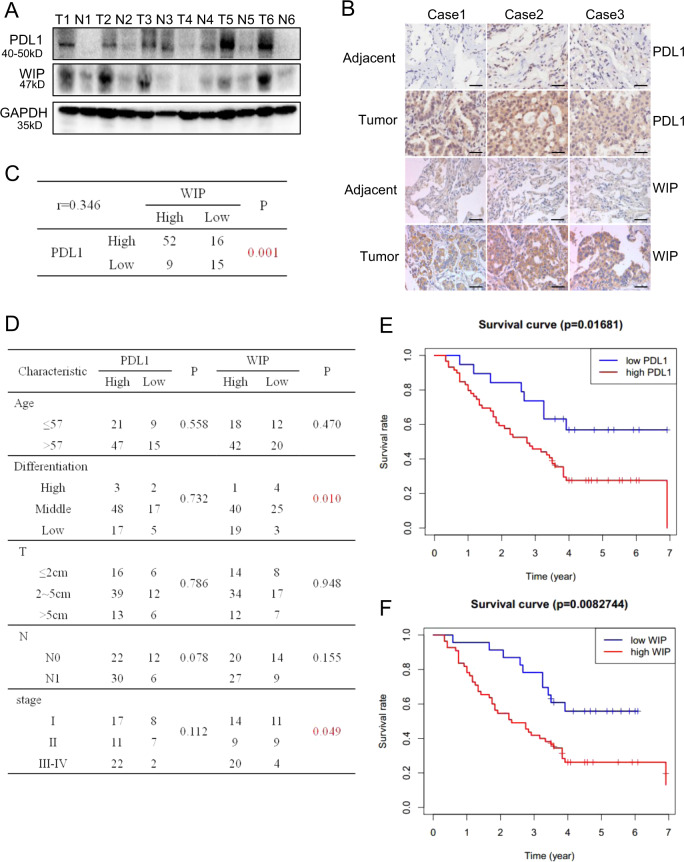
PD-L1 is positively correlated with WIP expression in lung adenocarcinoma tissues and predicts poor prognosis in patients. **a** PD-L1 and WIP expression of six human patient tissues and paired adjacent tissues was detected by Western blot. **b** PD-L1 and WIP expression from human lung adenocarcinoma tissue microarray of three cases with tumor and paired adjacent tissues was analyzed by immunostaining analysis. The representative images are shown. Scale bars = 100 μm. Original magnification: ×40. **c** The correlation between the expression of PD-L1 and WIP in human lung cancer tissues from 92 patients. **d** The relationship between the level of PD-L1 or WIP expression and clinicopathologic characteristics. **e**, **f** The relation between overall survival and expression of PD-L1 (*p* = 0.017) or WIP (*p* = 0.008) was analyzed by Kaplan–Meier analysis.

**Fig. 8:**
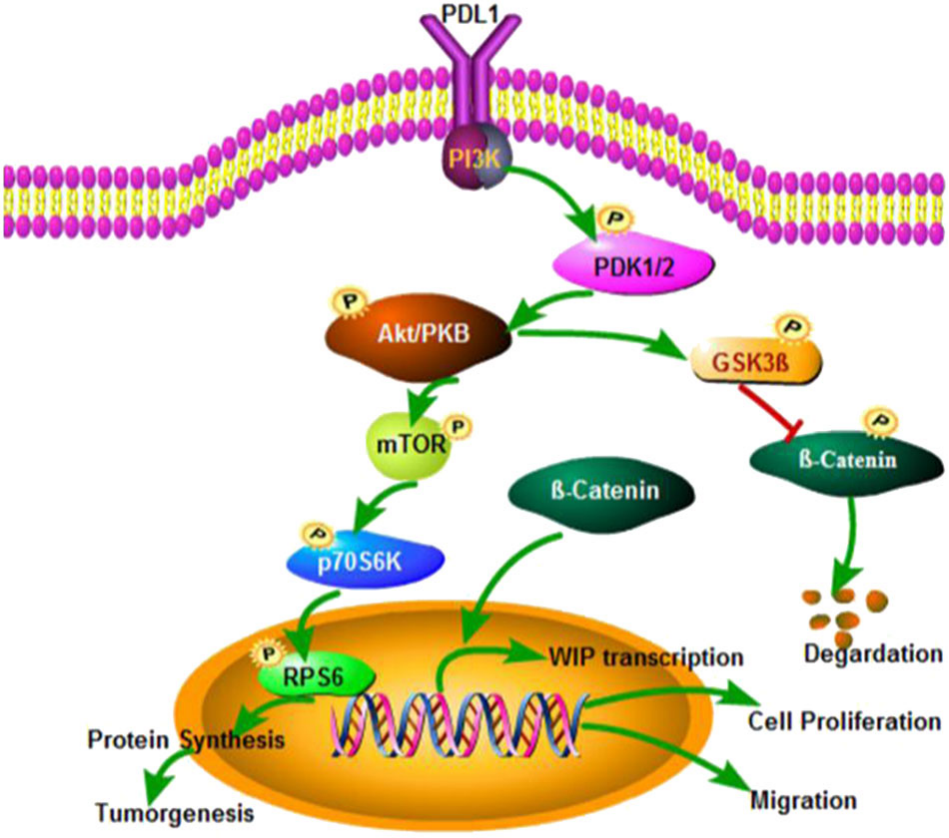
Schematic diagram illustrating the mechanism of PD-L1 involves in regulating lung cancer growth and progression. PD-L1 promotes p-S6 expression through PI3K/AKT/mTOR pathway, and also upregulates β-catenin expression by increasing phosphorylation of GSK3β (inactive form Ser9). β-Catenin binds to the WIP promoter and activates its expression in lung cancer cells.

We evaluated the expression of PD-L1 and WIP in human lung adenocarcinoma tissues (T) and paired adjacent normal tissues (N). As shown in Fig. 7a, the expression of PD-L1 and WIP was higher in patient tumor tissues (T) than adjacent normal tissues (N). We also analyzed the clinical significance of PD-L1 and WIP in 92 patients with lung adenocarcinoma based on tissue microarray. The immunohistochemistry staining of PD-L1 and WIP also showed high expression of PD-L1 and WIP in lung adenocarcinoma tumor tissues compared with the adjacent normal tissues (Fig. 7b). The positive correlation between PD-L1 and WIP expression in human lung adenocarcinoma tissues were observed (*p* = 0.001, *r* = 0.346) (Fig. 7c). Furthermore, the analysis of the clinocopathological data demonstrated that the expression of WIP was highly associated with lung tumor differentiation (*p* = 0.01) and TNM stage (*p* = 0.049) (Fig. 7d). The patients with high expression of PD-L1 and WIP had short survival time compared with those with low expression of PD-L1 and WIP (*p* = 0.017 and 0.008, respectively) (Fig. 7e, f). These results showed again the pro-tumorigenic function of the PD-L1 and WIP in human lung cancer progression (Fig. 8).

## Discussion

PD-L1 functions as an oncogene in several cancer types, including gastric cancer^7^, osteosarcoma^8^, esophageal cancer^13^, and ovarian cancer^12,21^. In our study, we found that PD-L1 promotes lung cancer cells proliferation, migration and invasion by activating PI3K/Akt/mTOR and Erk pathway. However, as a membrane and cytoplasma protein, how PD-L1 regulates the downstream signaling in cancers has not been fully understood. It has been reported that in melanoma PD-1 and PD-L1 interaction triggers ITIM and ITSM cytosolic loci of PD-1 to recruit phosphatases SHP-1 and SHP-2, which induces dephosphorylation of TCR in T cells, whereas promotes activation of pro-tumorigenic pathways in melanoma^29^. Furthermore, SHP-2 can positively regulate RTK signal transduction in glioma genesis^30,31^. NTRK1 (Neurotrophic Receptor Tyrosine Kinase 1), one of family member of RTKs, is downregulated by PD-L1 knockdown in our study. Whether the activation of PI3K/Akt pathway by PD-L1 is NTRK1 dependent should be further evaluated, and whether PD-1 and PD-L1 interaction is necessary for PD-L1 signaling transduction remains unclear.

Cell migration and invasion can be regulated by various factors. In our study, we have shown that PD-L1 promotes lung cancer cell migration and invasion by regulating EMT signaling. A previous study has reported that stathmin-1 can regulate trophoblast invasion. Decreased stathmin-1 expression inhibits trophoblast proliferation and invasion and is associated with recurrent miscarriage^32^. In addition, it has shown that TGF-β/smad pathway involves in tumor invasion and progression^33,34^. The activation of microRNA-520h–associated TGF-β1/c-Myb/Smad7 axis promotes epithelial ovarian cancer progression^33^. TGFβ can induce EMT by downregulating epithelial markers and upregulating mesenchymal markers^35^. However, whether TGF-β signaling is implicated in the PD-L1 mediated lung cancer invasion should be further clarified. The integrin/Erk pathway has been reported widely in both tumor invasion and pregnancy development. EIF5A1 promotes trophoblast migration and invasion via ARAF-mediated activation of the integrin/Erk signaling pathway^36^. In our study, we have also demonstrated that the Erk signaling plays an essential role in the PD-L1-mediated lung cancer proliferative promotion and invasion (Supplementary Fig. S3C, D). Furthermore, our analysis for RNA-Seq data also showed that the Ras/Erk signaling molecules were enriched in the negative control group compared with the PD-L1 siRNA-treated group (data not shown).

LncRNA HOX transcript antisense RNA (HOTAIR) is associated with cell invasion and tumorigenesis. Previous research have demonstrated that upregulation of PUM1 expression in preeclampsia decreases trophoblast invasion by negatively regulating the expression of the lncRNA HOTAIR^37^, and elevated tristetraprolin impairs trophoblast invasion in women with recurrent miscarriage by destabilization of HOTAIR^38^. Although HOTAIR has been shown to reduce in recurrent miscarriage, it elevates in brain metastasis and is positively correlated with poor prognosis in lung cancer^39^. Considering that HOTAIR can silence miR-34a^40^ and miR-34a inhibits PD-L1 expression in lung cancer^41^, the relationship between HOTAIR and PD-L1 needs be further investigated in lung cancer metastasis.

Our study also proved that PD-L1 promotes lung cancer cell proliferation at least partially through WIP both in vitro and in vivo. We could not exclude the possibility that other PD-L1 target genes also contributes to NSCLC cell growth. It has been reported that WIP mainly involves in actin cytoskeleton organization and polymerization that are required for the EMT^42^. However, how WIP affects NSCLC cells migration and invasion should be evaluated. Also, it would be significant to further determine whether PD-L1 and WIP promote metastasis of NSCLC in vivo.

The Hippo pathway and its regulation of YAP/TAZ are found to regulate organ size, tissue growth^43^ and cancer development^44^. This pathway is regulated principally by actin polymerization, which directly affects YAP/TAZ mediated transcription^45^. WIP also plays an essential role in actin skeleton organization. In our study, we found that PD-L1 negatively regulate the phosphorylation of YAP and promotes YAP nuclear translocation. Whether PD-L1 affects other proteins in Hippo pathway and WIP participates in this regulation should be further investigated.

We have proposed a model about the regulation of PD-L1 on lung cancer growth by activating Akt/mTOR and Erk pathway (Fig. 4c and Supplementary Fig. S3C). Our results showed that inhibition of PI3K/Akt, mTOR, and Erk pathway attenuates exogenous overexpression of PD-L1 (Fig. 4a, Supplementary Fig. S3A, B). This is consistent with the results that PD-L1 expression can be regulated via the PI3K/Akt and or Ras/MAPK pathways in different tumor cell types^46–48^.

Finally, we analyzed the expression of PD-L1 and WIP in human lung adenocarcinoma patients and mouse xenografts, we found that PD-L1 was mainly expressed in cytoplasm in 92 human lung adenocarcinoma tissues, which was not consistent with results of lung adenocarcinoma tissues that PD-L1 expressed in both membrane and cytoplasm^49^. In our data, only six patients were expressed in both cytoplasm and membrane (data not shown). In all mouse xenografts, PD-L1 was expressed in cytoplasm. Our work also revealed that PD-L1 expression was highly correlated to WIP expression in 92 human lung adenocarcinoma patients. High expression of both PD-L1 and WIP in human lung adenocarcinoma was associated with patient poor survival, which was consistent with previous study in other types of malignancy, such as esophageal cancer^13^, breast cancer^19^, and pancreatic ductal adenocarcinoma^26^.

In conclusion, we have revealed a new oncoprotein expression profile of WIP in NSCLC and demonstrated that PD-L1 regulated proliferation and migration of NSCLC cells via Akt-β-catenin-WIP axis. Our results provide new insights into understanding the pro-tumorigenic role of PD-L1 and its regulatory mechanism on WIP in lung cancer, and suggest that the PD-L1/Akt/β-catenin/WIP signaling axis may be a potential therapeutic target for lung cancers.

## Supplementary information

supplementary figure legends

supplementary figure 1

supplementary figure 2

supplementary figure 3

supplementary figure 4

supplementary figure 5
