# Supplementary material for: PD-L1 promotes tumor growth and progression by activating WIP and β-catenin signaling pathways and predicts poor prognosis in lung cancer
Source: Cell Death Dis. 2020 Jul 6;11(7):506. doi: 10.1038/s41419-020-2701-z (PMC7338457; doi:10.1038/s41419-020-2701-z)
Supplement: Supplementary file 1 — supplementary figure legends [file 41419_2020_2701_MOESM1_ESM.docx]

**Supplementary Figure Legends**

**Figure S1. PD-L1 regulates cell viability and tumor growth in lung cancer.** (A) PD-L1 was knocked down in H460 and H358 cells and overexpressed in H1299 and A549 cells. (B) Cell viability was measured by MTT assay. The data represent the mean ± SD of three independent experiments, *P<0.05, **P<0.01, ***P<0.001. (C) The expression of PD-L1 protein from BALB/c-Nude xenografts was measured by Western blotting. (D) The expression of PD-L1 in BALB/c-Nude xenograft tissues was measured by immunohistochemistry stain. Scale bars=50μm. Original magnification: ×40.

**Figure S2. PD-L1 overexpression and knockdown do not affect stemness characters and apoptosis.** The expression of CD44, OCT4, Bax, Bcl-2 in human lung A549, H1299 and H460 cells were detected by Western blotting.

**Figure S3. The mTOR and Erk signaling pathways are involved in the proliferative and invasive promotion of lung cancer cells mediated by PD-L1.** (A) H1299 and H358 cells were pre-treated with 100 nM rapamycin for 4 hours and then cells were transfected with PDL1-OE plasmid or siRNA for 48h. The expression of p-P70S6K and p-S6 were detected by Western blotting. (B) Cells were pre-treated with 10 uM U0126 and then transfected with plasmid or siRNA. The expression of ERK, p-ERK, p-P70S6K, p-S6 were detected by Western blot. (C-D) Cells were pre-treated with U0126 at 10 uM for 4 hours and then transfected with PDL1-OE plasmid or siRNA for 48h. Cell proliferation and invasion were analyzed by MTT and transwell invasion assay. The data represent the mean ± SD of three independent experiments, *P<0.05, **P<0.01, ***P<0.001. NS means No significant differences between two groups. U means U0126.

**Figure S4. PD-L1 regulates β-catenin and its downstream genes.** (A) Cells were transfected with PDL1-OE plasmid or siRNA for 48 hours, and β-catenin mRNA levels were abalyzed by RT-PCR. (B) The potential transcriptional binding sites of β-catenin on the WIP promoter. (C) Cells were transfected with PDL1-OE plasmid or siRNA for 48 hours, and the protein expression of Cyclin D1, p-YAP and total YAP were detected by Western blot. (D) Cells were transfected with PDL1-OE plasmid or siRNA for 48 hours, and the subcellular localization of YAP in lung cancer cell was analyzed by immunofluorescence staining assay. Scale bars=50μm.

**Figure S5. Full blot imformation for all gels in Figure 5C.**
