# Supplementary figures and images for: PD-L1 promotes tumor growth and progression by activating WIP and β-catenin signaling pathways and predicts poor prognosis in lung cancer
Source: Cell Death Dis. 2020 Jul 6;11(7):506. doi: 10.1038/s41419-020-2701-z (PMC7338457; doi:10.1038/s41419-020-2701-z)

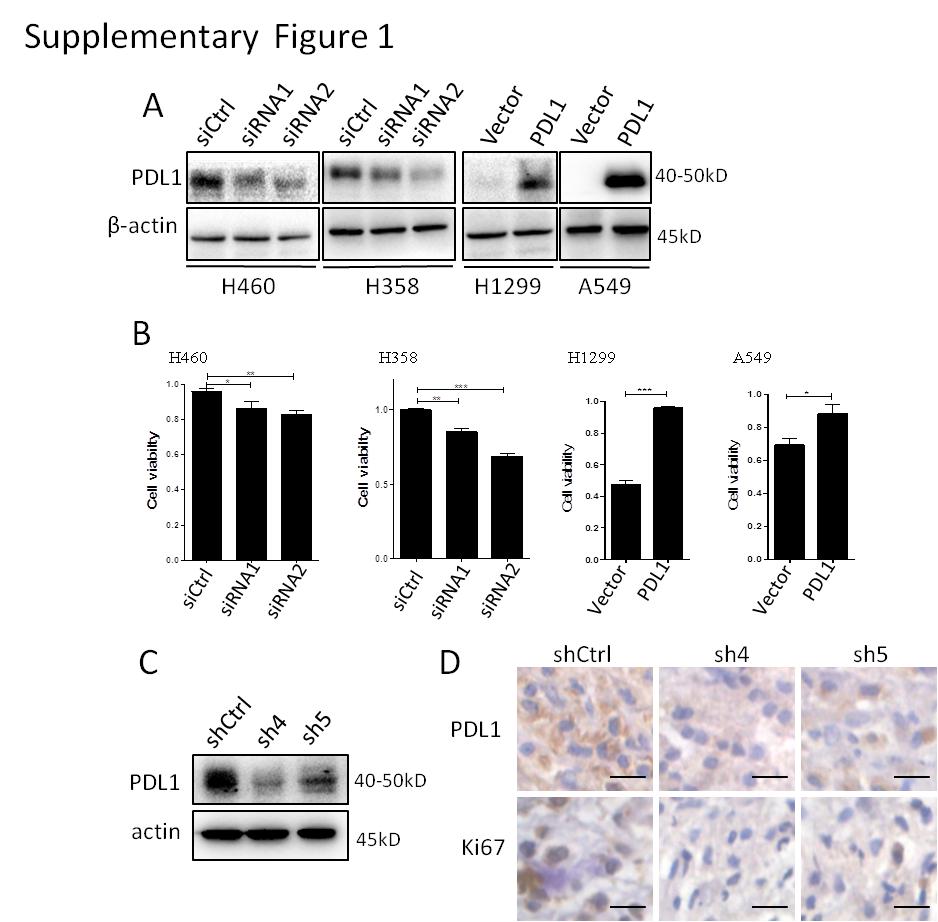

Supplement: Supplementary file 2 — supplementary figure 1 [file 41419_2020_2701_MOESM2_ESM.tif]

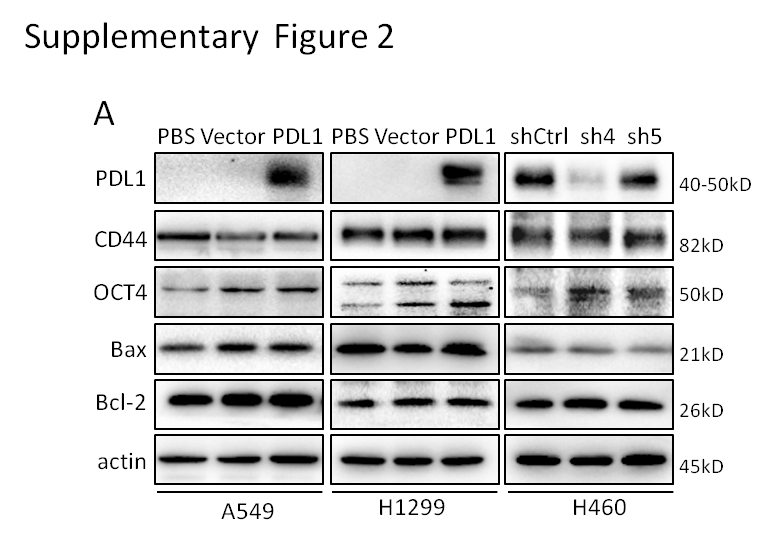

Supplement: Supplementary file 3 — supplementary figure 2 [file 41419_2020_2701_MOESM3_ESM.tif]

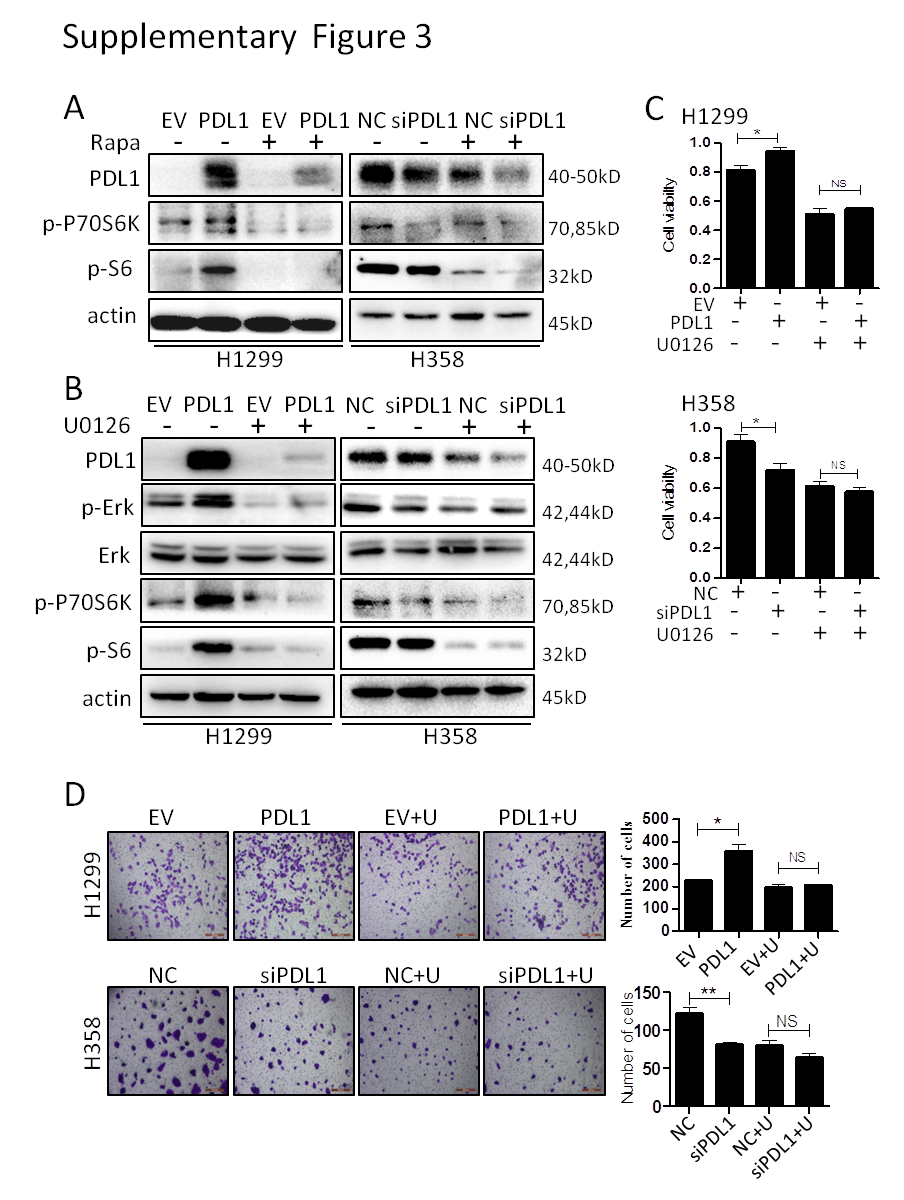

Supplement: Supplementary file 4 — supplementary figure 3 [file 41419_2020_2701_MOESM4_ESM.tif]

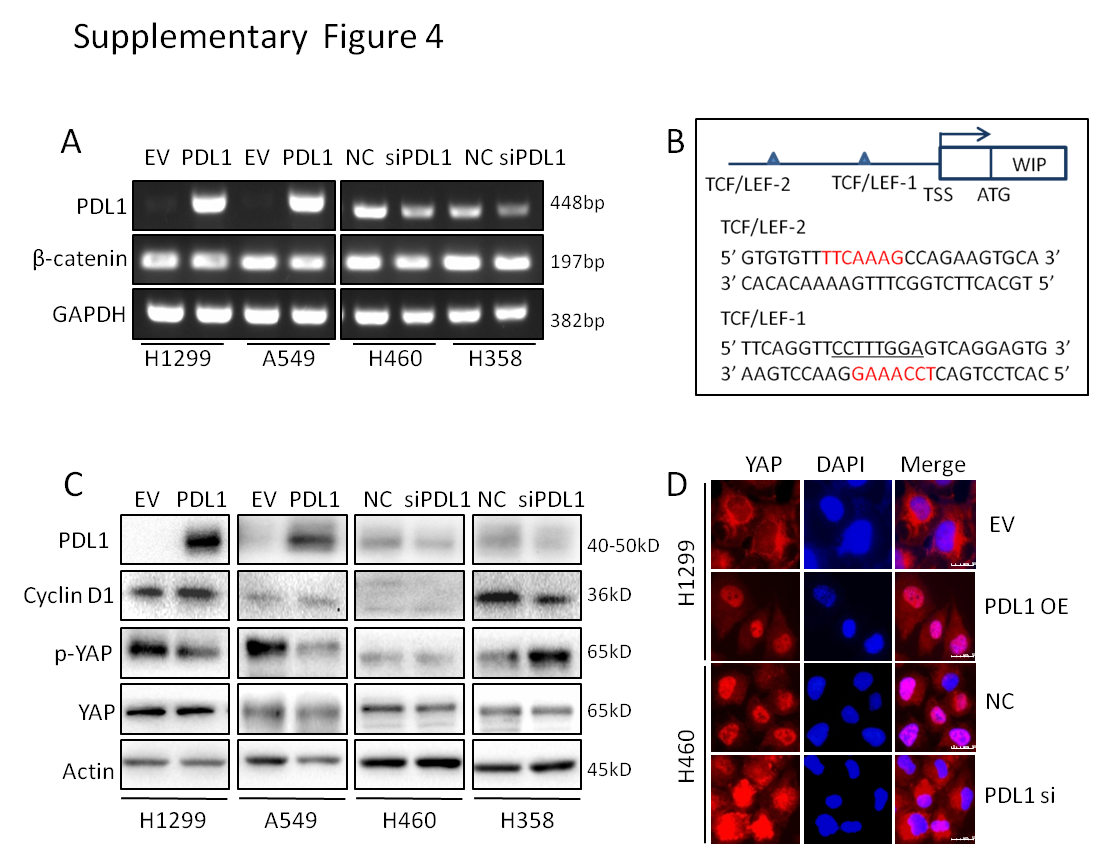

Supplement: Supplementary file 5 — supplementary figure 4 [file 41419_2020_2701_MOESM5_ESM.tif]

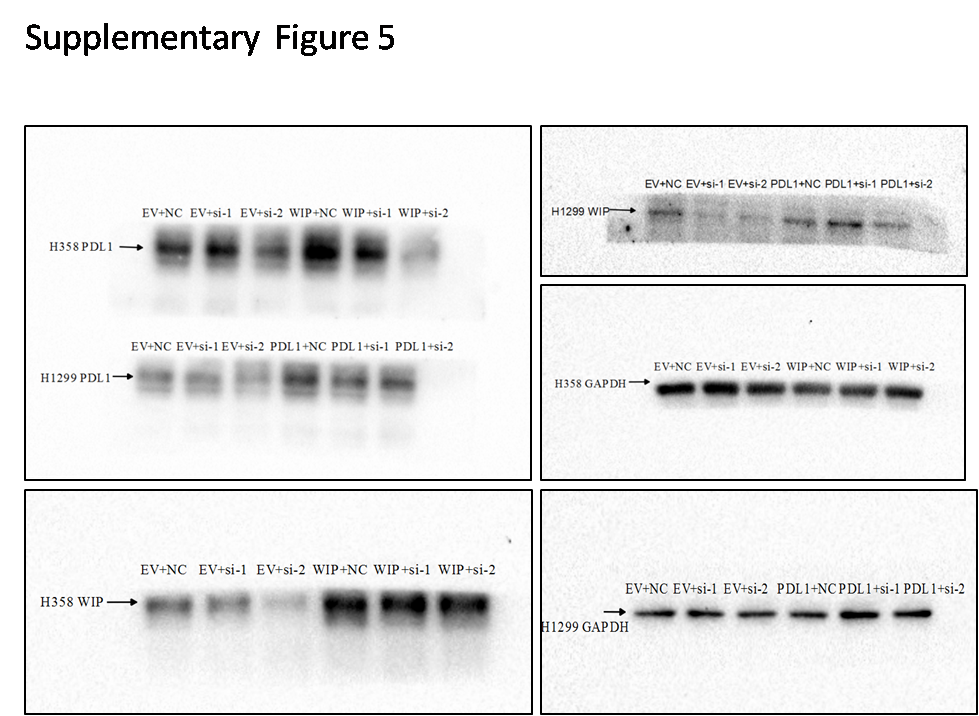

Supplement: Supplementary file 6 — supplementary figure 5 [file 41419_2020_2701_MOESM6_ESM.tif]
